# Performance of Flow Cytometry-Based Rapid Assay in Detection of Carbapenemase-Producing Enterobacterales

**DOI:** 10.3390/ijms25147888

**Published:** 2024-07-18

**Authors:** Blanca Pérez-Viso, Inês Martins-Oliveira, Rosário Gomes, Ana Silva-Dias, Luísa Peixe, Ângela Novais, Cidália Pina-Vaz, Rafael Cantón

**Affiliations:** 1 Servicio de Microbiología, Hospital Universitario Ramón y Cajal and Instituto Ramón y Cajal de Investigación Sanitaria (IRYCIS), 28034 Madrid, Spain; blancapv45@gmail.com; 2 FASTinov, S.A., 4200-135 Porto, Portugal; ioliveira@fastinov.com (I.M.-O.); rosariogomes@fastinov.com (R.G.); anadias@fastinov.com (A.S.-D.); 3 Division of Microbiology, Department of Pathology, Faculty of Medicine, University of Porto, 4200-319 Porto, Portugal; 4 CINTESIS–Center for Health Technology and Services Research, Faculty of Medicine, 4200-450 Porto, Portugal; 5 UCIBIO-Applied Molecular Biosciences Unit, Department of Biological Sciences, Faculty of Pharmacy, University of Porto, 4050-313 Porto, Portugal; lpeixe@gmail.com (L.P.); angelasilvanovais@gmail.com (Â.N.); 6 Associate Laboratory i4HB-Institute for Health and Bioeconomy, Faculty of Pharmacy, University of Porto, 4050-313 Porto, Portugal; 7 CIBER de Enfermedades Infecciosas, Instituto de Salud Carlos III, 28029 Madrid, Spain

**Keywords:** carbapenemases, Enterobacterales, antimicrobial resistance mechanisms, flow cytometry

## Abstract

Carbapenemase-producing Enterobacterales are increasingly being recognized in nosocomial infections. The performance of a flow cytometry-based rapid assay for their detection and differentiation was evaluated. This is a disruptive phenotypic technology, phenotypic and growth-independent, that searches for the lesions produced by drugs acting on cells after a short incubation time. Overall, 180 Gram-negative bacteria were studied, and results were compared with those obtained molecularly by PCR and phenotypically by ‘KPC, MBL and OXA-48 Confirm Kit’. This phenotypic method was used as reference for comparison purposes. Susceptibility to carbapenems (imipenem, meropenem, and ertapenem) was determined by standard broth microdilution. Overall, 112 isolates (62.2%) were carbapenemase producers, 41 KPCs, 36 MβLs, and 31 OXA-48, and 4 strains were KPC + MβL co-producers. Sixty-eight isolates were carbapenemase-negative. The percentage of agreement, sensitivity, and specificity were calculated according to ISO 20776-2:2021. The FASTinov assay showed 97.7% agreement with the reference method for carbapenemase detection. Discrepant flow cytometry results were obtained in four isolates compared with both reference and PCR results. The sensitivity and specificity of this new technology were 95.3% and 98.5%, respectively, for KPCs, 97.6% and 99.3% for MβLs, and 96.9% and 98% for OXA-48 detection. In conclusion, we describe a rapid flow cytometry assay with high accuracy for carbapenemase detection and the differentiation of various carbapenemases, which should impact clinical microbiology laboratories and patient management.

## 1. Introduction

Antimicrobial resistance (AMR) is a global health problem declared to be one of the top 10 public health threats facing humanity [1]. In 2019, 4.95 million deaths were associated with bacterial antibiotic resistance, and 1.27 million deaths were directly attributable to bacterial AMR [2]. Carbapenemase-producing Enterobacterales (CPEs) are increasingly recognized in nosocomial infections, including *Klebsiella pneumoniae* and many AmpC producers such as *Enterobacter* spp. and *Serratia marcescens* [3]. The World Health Organization (WHO)’s 2024 global priority list of pathogens ranks carbapenem-resistant *Acinetobacter baumannii*, carbapenem-resistant Enterobacterales, and carbapenem-resistant *Pseudomonas aeruginosa* in the highest priority critical category [4].

The most relevant mechanism of carbapenem resistance in Gram-negative infections is the production of carbapenemases (enzymes able to hydrolyse antibiotics), mainly encoded in highly transmissible plasmids [5]. These enzymes represent one of the most versatile families of β-lactamases with a broad spectrum of activity. Regarding the Ambler classification [6], they are grouped into four classes, and carbapenemases belong to three of them: A, B, and D. Class A enzymes includes the prevalent *K. pneumoniae* carbapenemases (KPCs), class B includes the VIM IMP and the New Delhi NDM metallo-β-lactamases (MβLs), among others, and class D includes oxacillinase OXA-48-like carbapenemases. Class C includes AmpC β-lactamases that are not carbapenemase per se, but they can play an important role in carbapenem resistance when combined with other resistance mechanisms [5]. These enzymes are globally distributed but found unequally in different geographic areas. KPCs are highly frequent worldwide; MβLs have been mostly associated with some countries in Asia and specific European countries, and OXA-48 is associated with Mediterranean countries [7].

Rapid phenotypic susceptibility assays are essential for accurate and swift therapeutic decisions, and several new methods have recently received CE marking [8]. FASTinov, a spinoff from the University of Porto (Portugal), has developed and validated two kits, FASTgramneg and FASTgrampos, to determine antimicrobial susceptibility with great accuracy directly from positive blood cultures using flow cytometry [9]. Unlike most antimicrobial susceptibility test technologies, this innovative approach is not growth-dependent. Flow cytometry, commonly used in clinical laboratories of haematology and human cell studies, is employed here to analyse bacterial cells after a brief incubation period (1 h) with the antibiotics. The automated system evaluates around 30,000 cells within seconds, recording multiple parameters such as fluorescence intensity, size, and complexity. Treated cells are compared to untreated controls, and proprietary software reports susceptibility (if changes are observed) or resistance (if treated cells are similar to controls). Additionally, this approach has been used to identify various resistance mechanisms, such as detecting ESβL, carbapenemases [KPC, MβL, and OXA-48], and pAmpC enzymes [10]. Flow cytometry together with a computational approach was also used to study the synergic effect between carbapenems [11].

In this article, we described a rapid flow cytometry-based assay with a time-to-result of up to 2 h, with the main objective being not only to detect carbapenemases but also to classify different class A, B, and D carbapenemases in a collection of Enterobacterales isolates that screened positive for carbapenemase production according to the EUCAST protocol for the detection of mechanisms of resistance (Minimum Inhibitory Concentration–MIC–for meropenem > 0.125 mg/L) [12]. Discrimination of the carbapenemase type (e.g., KPC, NDM, VIM, or OXA-48, among others) is relevant for the selection of antimicrobial treatment, as they are differently inhibited by the β-lactamase inhibitors present in current combinations with β-lactams. The selection of appropriate β-lactam–β-lactamase inhibitor combinations in infections due to carbapenemase-producing microorganisms have implications in patients’ outcomes [13,14].

## 2. Results

A series of 180 Enterobacterales, which were previously well characterized and with meropenem MICs > 0.125 mg/L and/or resistance to at least one carbapenem, were selected among different clinical isolate collections from FASTinov S.A, a spinoff of the University of Porto, Portugal, CCP—Culture Collection of Porto—Faculty of Pharmacy University of Porto, Portugal, and from the Microbiology Department at Ramón y Cajal Hospital in Madrid, Spain. Species identification distribution is shown in Table 1, with *K. pneumoniae* (31.6%, 57/180) and *S. marcescens* (20%, 36/180) being the most prevalent in this study.

**Figure 1:**
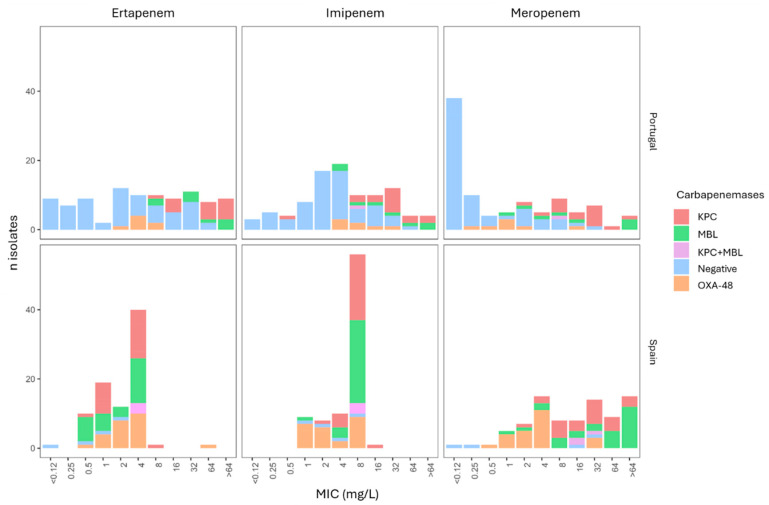
Value distribution of carbapenem minimum inhibitory concentration (MIC, mg/L) and carbapenemase production of isolates in the studied Enterobacterales collection.

The susceptibility to different carbapenems is detailed in Table 2, and the distribution of different carbapenemases according to ertapenem (ERT), imipenem (IMI), and meropenem (MRP) MIC values is represented in Figure 1. Overall, 17 isolates were susceptible to all carbapenems; 15 of them were also negative for the carbapenemase screening (meropenem MIC > 0.125 mg/L) recommended by the European Committee of Antimicrobial Susceptibility Testing (EUCAST) [14]. Meropenem exhibited a MIC > 0.12 mg/L MIC in a total of 39 isolates.

Phenotypic carbapenemase characterization results by ‘KPC, MBL, OXA-48 Confirm Kit’, based on meropenem and different carbapenemase inhibitors (boronic acid for KPCs, EDTA for MβLs, and temocillin for OXA-48), showed that 41 strains were KPC producers (25 from Spain, 16 from Portugal), 36 were MβL producers (28 from Spain, 8 from Portugal), and 31 were OXA-48 producers (24 from Spain, 7 from Portugal). A total of 4 isolates co-produced two different carbapenemases (KPC + MβL) (three from Spain, one from Portugal), and 68 were negative for the production of carbapenemase (64 from Portugal, 4 from Spain); 15 of them were the wild-type for carbapenems, 3 were resistant to all carbapenems, and 50 exhibited resistance at least to one carbapenem.

PCR and Sanger sequencing confirmed these results in total agreement with phenotypic results. *bla*_VIM-1_ (n = 31), *bla*_OXA-48_ (n = 31), and *bla*_KPC-2_ (n = 25) genes were the most prevalent ones, and the co-production of *bla*_NDM-1_ + *bla*_KPC-2_ (n = 1), *bla*_VIM-1_ + *bla*_KPC-3_ (n = 1), and *bla*_VIM-1_ + *bla*_KPC-2_ (n = 2) was also reported.

Regarding phenotypic flow cytometry assay results (with the same rationale of phenotypic reference method), 68 isolates were negative in carbapenemase production (64 from Portugal and 4 from Spain). The corresponding values for carbapenemase production were 112 isolates (80 from RyC, Spain, and 32 from Portugal). Within carbapenemase producers, 34 isolates were classified as KPC producers due to an increase in fluorescence in the presence of meropenem plus boronic acid (18 from Spain, 16 from Portugal), and 32 strains were classified as MβL producers with an increase in fluorescence in the presence of meropenem plus EDTA (24 from Spain, 8 from Portugal). In addition, 33 strains were classified as OXA-48 producers with a low intensity of fluorescence with temocillin (resistance) and an increase in fluorescence intensity in the presence of meropenem and different carbapenemase inhibitors (26 from Spain, 7 from Portugal). Four strains (three from Spain, one from Portugal) were classified as double-carbapenemase producers, KPC + MβL, due to an increase in fluorescence in the presence of meropenem plus boronic acid and with meropenem plus EDTA in combination. This coproduction was also confirmed by PCR. Of note is that in nine cases (all from Madrid), five KPC-producing isolates and four MβL-producing isolates were detected and identified by both phenotypic and molecular methods; flow cytometry was able to detect only the presence of carbapenemase production but was not able to discriminate between KPC and MβL production (Table 3). In all of these isolates, a positive increase in fluorescence was detected in wells in which meropenem with both boronic acid and EDTA were included, but not in wells in which each inhibitor individually was combined with meropenem. These results were not considered discrepant, as carbapenemase production was detected (despite no type discrimination), although further confirmation with other methods was needed. Discrepant results (*) comparing flow cytometry with our phenotypic reference method and PCR molecular testing were reported in four isolates (Table 3).

The FASTinov flow cytometry assay showed a 97.8% agreement with the RM. Note that the nine isolates classified as having KPCs or MβLs were not considered errors, as flow cytometry was able to detect carbapenemase but was not able to discriminate the carbapenemase class (Table 3). Taking these results into account, the sensitivity and specificity of the flow cytometry assay compared with the RM were 95.3% and 98.5%, respectively, for KPCs, 97.6% and 99.3% for MβLs, and 96.9% and 98% for OXA-48 detection.

**Figure 2:**
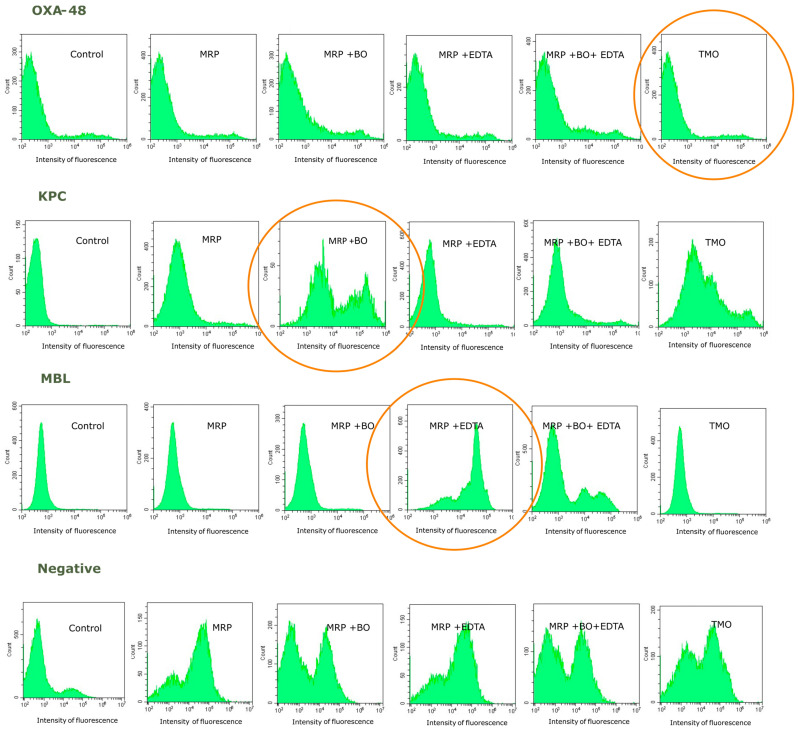
Histograms representing different examples of carbapenemase-producing isolates. MRP, meropenem (2 mg/L); MRP + BO, meropenem + boronic acid (2 + 5 mg/L); MRP + EDTA, meropenem + EDTA (2 + 1.25 mg/L); MRP + BO + EDTA, meropenem + boronic acid + EDTA (2 + 5 + 1.25 mg/L); TMO, temocillin (64 mg/L); and OXA-48-producing *K. pneumoniae*. Low intensity of fluorescence (resistance) is shown after 1 h of incubation with temocillin, whereas no significant increase in the intensity of fluorescence was observed when the strain was incubated with meropenem plus boronic acid and EDTA carbapenemase inhibitors; KPC-producing *E. cloacae*, in which cytometry showed an increase in the fluorescence intensity when the strain was incubated with meropenem and boronic acid (orange circles); MβL-producing *K. pneumoniae* in which an increase was observed after the incubation with meropenem and EDTA; and negative carbapenemase-producing isolate (susceptible to meropenem).

An example of flow cytometry analysis for carbapenemase detection is shown in Figure 2.

## 3. Discussion

Bacterial infections caused by CPE have become a global public health problem. Carbapenemases are widely reported worldwide and are associated with an increase in mortality in patients with CPE infections compared with non-CPE-infected people [7]. From a clinical point of view, the early detection of specific carbapenem resistance mechanisms is critical to reduce a patient’s mortality, length of hospitalization, and associated costs, as the patient will receive correct targeted treatment, which will also depend on the carbapenemase type [15].

Flow cytometry has been proven to be an accurate and rapid technique to determine antimicrobial susceptibility and detect resistance mechanisms such as ESβL, carbapenemases, and/or AmpC [10]. Regarding carbapenemase detection, lesions produced by drugs are detected by a flow cytometer after the bacteria are stained with a fluorescent probe. A synergistic effect is observed when a carbapenem (meropenem) associated with inhibitors (boronic acid or EDTA) shows an increase in the intensity of fluorescence compared to the carbapenem alone. In addition, molecular assays are a rapid alternative to phenotypic tests, although discrepancies between both methods have been reported [16].

According to the prospective multinational European Survey on Carbapenemase-Producing Enterobacterales (EuSCAPE), it is known that available treatment options with safe antibiotics still lack effectiveness [17]. Regarding the EuSCAPE’s results, 37% of carbapenem-resistant *K. pneumoniae* and 19% of carbapenem-resistant *E. coli* were confirmed to harbour carbapenemase genes, the most frequent being those encoding KPCs (42%) and OXA-48 (38%) carbapenemases. However, 29.3% (353/1203) of *K. pneumoniae* and 60.3% (117/194) of *E. coli* isolates were confirmed to also present other resistance mechanisms. Moreover, an increase in Enterobacterales isolates co-producing different carbapenemases has been reported in Europe and also in other geographic areas such as South America [18].

Phenotypic tests detecting resistance mechanisms are part of the routine work in clinical microbiology laboratories. For carbapenemases, these methods are based on the expression of carbapenemase enzymes during bacterial growth (i.e., up to 24–48 h), using imipenem or meropenem as a substrate. In addition, other methods such as those based in colorimetric (e.g., Carba NP) or in immunochromatography assays are also recommended. GeneXpert, Carba-R platform, or BioFire FilmArray are some examples of rapid molecular tests also used for carbapenemase detection. All these methods are useful to improve CPE surveillance and patients’ treatment [12,19,20,21].

Regarding molecular and biochemical rapid tests for carbapenemase detection, there are some points that need to be considered. A negative test does not always mean that the organism is carbapenem-susceptible (resistance could be associated with non-enzymatic mechanisms). Furthermore, resistance gene level expression should be taken into account because the presence of a gene does not necessarily mean the organism is resistant to carbapenems. Additionally, biochemical tests will not determine the specific carbapenemase enzyme [19]. The distinction of different carbapenemases is necessary to implement current guidelines addressing the treatment of infections due to carbapenemase-producing microorganisms [13,14]. The type of carbapenemase (e.g., KPC, VIM, NDM, or OXA-48, among others) determines the selection of the antimicrobial treatment, as they are differently inhibited by the β-lactamase inhibitors that are associated with β-lactams.

In our study, flow cytometry allowed us to accurately identify the presence of carbapenemases with 97.8% agreement for the most important carbapenemase families, although the principle could be extended to other ones. Moreover, one of the most remarkable advantages of flow cytometry is the time-to-result, which was 15 min per isolate and did not require overnight culture. Nevertheless, there were nine strains in which the presence of KPCs or MβLs could not be discriminated, and the intensity of fluorescence with both inhibitors (boronic acid, 5 mg/L and EDTA, 1.25 mg/L) showed a synergic effect with meropenem; therefore, distinction between the presence of KPC or MβL enzymes was not possible (Table 3). It is important to note that phenotypic resistance was nevertheless detected in these cases, although other inhibitor concentrations may need to be tested in order to increase the specificity of the method. It is important to note that false negative results were not obtained; the cytometer was able to detect carbapenemase production in all isolates in which the reference method was also positive. The developed flow cytometry protocol needs further validation in other laboratories using a higher number of isolates, especially the most challenging ones. Although flow cytometry is common in haematology and immunology laboratories, it is not as widely present in the clinical microbiology field. In that sense, a collaboration with the Ramón y Cajal Hospital in Madrid, through the European project FAST-bact, facilitated the validation of the developed protocols and provided valuable feedback for future intended users with great results regarding sensitivity, specificity, and global performance [9] as well as concrete antimicrobial susceptibility determination such as ceftolozane-tazobactam [22].

In conclusion, in this study, a rapid (2 h) and phenotypic flow cytometry-based assay for the detection and discrimination of different carbapenemase types was described with great accuracy. This approach could have a great impact in the clinical microbiology setting, both for patients’ treatment and for the epidemiological control of resistance mechanisms.

## 4. Material and Methods

### 4.1. Study Design, Sample Collection and Carbapenemase Detection

Overall, 180 non-duplicated Gram-negative bacilli, belonging to different well-characterized clinical collections, were studied in a two-site study (53 from FASTinov S.A, a spinoff of the University of Porto, Portugal, 43 from CCP—Culture Collection of Porto—Faculty of Pharmacy, University of Porto, and 84 isolates from the Microbiology Department at Ramón y Cajal Hospital in Madrid, Spain). Different species of Enterobacterales (other than *K. pneumoniae* and *E. coli*) were included in order to have a diverse representation of the isolates that can be found in the hospital environment. Both the period of isolation and the source were diverse. The only inclusion criteria were that isolates presented a meropenem MIC screening cut-off value > 0.125 mg/L (n = 141) and/or that they exhibited resistance to at least one carbapenem IMI, MRP, or ERT (n = 163). Also, 15 isolates with an MRP-negative screening and susceptibility to all carbapenems were also included in this study in order to evaluate the flow cytometry kit’s performance.

**Figure 3:**
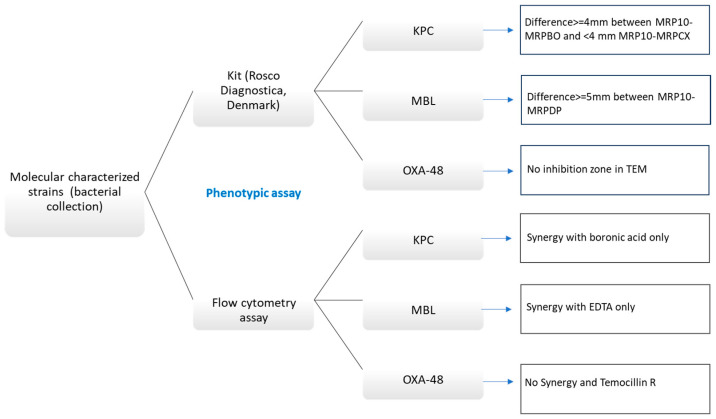
Description of the algorithm for carbapenemase detection by phenotypic methods; Rosco Diagnostica kit (disk diffusion reference method) and flow cytometry; MRP10 (meropenem 10 μg); MRPBO (meropenem 10 μg + boronic acid); MRPCX (meropenem 10 μg + cloxacillin); MRPDP (meropenem 10 μg + dipicolinic acid); and TEM (temocillin 30 μg).

Carbapenem MIC determination was performed using broth microdilution in accordance with ISO 20776-2:2021 [23], and the results were interpreted according to EUCAST 2024 breakpoints (https://www.eucast.org/clinical_breakpoints, accessed on 12 July 2024). Carbapenemase production was phenotypically confirmed using the commercial ‘KPC, MBL, OXA-48 Confirm Kit’ (Rosco Diagnostica, Taastrup, Denmark) as a reference method (Figure 3). The detection of genes encoding carbapenemases was performed by PCR using primers and conditions previously described [24] and further nucleotide Sanger sequencing.

### 4.2. Flow Cytometry and Data Analysis

Carbapenemase detection was also performed with FASTinov flow cytometry technology in order to detect carbapenemase production and discriminate between KPCs, MβLs, and OXA-48 (Figure 3). To accomplish this, the kit included meropenem alone and in combination with different carbapenemase inhibitors. Four controls were used: *K. pneumoniae* ATCC 700603, an ESβL producer, as the negative control, *K. pneumoniae* BAA 1705 (KPC producer), *K. pneumoniae* NCTC 13443, and *E. coli* NCTC 13476 (MβL producers) as positive controls.

The FASTinov flow cytometry kit comprised a 96-well microplate that contained meropenem and carbapenemase inhibitors (boronic acid for KPCs, EDTA for MβLs, and temocillin for OXA-48) at different concentrations, a fluorescent dye, the CytoFLEX flow cytometer from Beckman Coulter (Brea, CA, USA), and bioFAST 2.0 (FASTinov, Porto, Portugal), and dedicated software able to interpret cytometry results.

To inoculate the FC panel, bacterial colonies previously grown in blood agar plates were used. The procedure was as follows: (1) Two isolated colonies were picked from the plate and inoculated in 5 mL of brain heart broth. (2) This suspension was incubated at 37 °C on an orbital shaker (500 rpm) for at least 1 h or until evident growth was observed. (3) The suspension was centrifuged at 5000 rpm for 5 min. The supernatant was discarded, and 2 mL of sterile saline solution was added. (4) The inoculum was adjusted to 0.5 McFarland and diluted (1:100) in filtered cation-adjusted Mueller–Hinton II broth.

Each suspension was inoculated (100 µL) in a 96-well microplate panel previously prepared with 2 mg/L or 8 mg/L of meropenem alone or associated with EDTA for MβL detection, boronic acid for KPC detection, and/or temocillin for OXA-48 detection; also, isolates were exposed to both inhibitors together (boronic acid + EDTA). A membrane depolarization fluorescent dye was added to each well. After 1 h of incubation in darkness and shaking at 37 °C, the panel was analysed in a CytoFLEX flow cytometer to compare cells with and without inhibitors. Proprietary software called bioFAST, also developed by FASTinov, analysed each experiment, taking into account different parameters such as cell fluorescence intensity, number of events, and morphological cell changes, in order to phenotypically determine the production of carbapenemase enzymes. Discrepant results between the reference method and flow cytometry were confirmed with disk diffusion.

### 4.3. Statistical Analysis

Cut-off values for flow cytometry were calculated using ROC curves and were introduced in bioFAST SW in order to detect the presence of carbapenemases according to EUCAST breakpoints. The percentage of agreement, sensitivity, and specificity of the performance of the cytometry assay were determined according to ISO 20776-2:2021 using phenotypic characterization as the reference method.

## Figures and Tables

**Table 1:** Species distribution regarding sites of study: Portugal and Spain.

Microorganisms	Portugal	Spain	Total
*Citrobacter freundii*	4	3	7
*Citrobacter koseri*	0	1	1
*Enterobacter asburiae*	3	12	15
*Enterobacter cloacae*	1	12	13
*Enterobacter hormaechei*	14	0	14
*Enterobacter kobei*	3	9	12
*Escherichia coli*	9	1	10
*Klebsiella aerogenes*	8	0	8
*Klebsiella oxytoca*	1	0	1
*Klebsiella pneumoniae*	44	13	57
*Kluyvera cryoscescens*	1	0	1
*Morganella morganii*	3	0	3
*Proteus mirabilis*	0	1	1
*Providencia stuartii*	1	0	1
*Serratia marcescens*	4	32	36
Total	96	84	180

**Table 2:** Carbapenem susceptibility profiles of all studied isolates (n = 180).

	ETP	IMI	MRP
R	144 (80)	126 (70)	85 (47.2)
I	0 (0)	0 (0)	16 (8.9)
S	36 (20)	54 (30)	79 (43.9)

EUCAST criteria (2024). ETP, ertapenem; IMI, imipenem; MRP, meropenem; S, susceptible; I, susceptible, increased exposure; R, resistant. Data expressed as n (percentage).

**Table 3:** Comparison of flow cytometry results with those obtained with the reference method and PCR in isolates with different results when using different methods.

MIC (mg/L)						
Isolate	Phenotype (RM)	PCR	FC	IMI	MRP	ERT
*C. freundii*	KPC	KPC-2	KPC/MβL	8	4	1
*E. asburiae*	KPC	KPC-2	KPC/MβL	8	32	4
*E. asburiae*	KPC	KPC-3	KPC/MβL	8	16	4
*E. kobei*	KPC	KPC-2	KPC/MβL	8	8	4
*S. marcescens*	MβL	VIM-1	KPC/MβL	8	2	4
*S. marcescens*	MβL	VIM-1	KPC/MβL	8	16	4
*S. marcescens*	MβL	VIM-1	KPC/MβL	8	>64	0.5
*S. marcescens*	KPC	KPC-2	KPC/MβL	16	8	4
*S. marcescens*	MβL	VIM-1	KPC/MβL	8	>64	4
*C. freundii*	KPC	KPC-2	OXA-48 *	4	16	4
*C. koseri*	KPC	KPC-2	OXA-48 *	4	>64	4
*S. marcescens*	MβL	VIM-1	OXA-48 *	8	>64	0.5
*E. asburiae*	OXA-48	OXA-48	MβL *	1	4	4

MIC: minimum inhibitory concentration; RM: reference method, ‘KPC, MβL, OXA-48 Confirm Kit’; PCR: polymerase chain reaction; FC: flow cytometry; IMI: imipenem; MRP: meropenem; ERT: ertapenem; *: discrepant results.

## Data Availability

All data are contained within this article; additional information can be requested to the corresponding authors.
